# Effect of UV-C Treatment on Shelf Life of Soft Wheat Bread (Bun)

**DOI:** 10.3390/foods13060949

**Published:** 2024-03-20

**Authors:** Rita Chiara Romano, Cristina Restuccia, Chiara Alessandra Carmen Rutigliano, Santi Spartà, Lucia Parafati, Riccardo N. Barbagallo, Giuseppe Muratore

**Affiliations:** 1 Dipartimento di Agricoltura, Alimentazione e Ambiente (Di3A), University of Catania, Via S. Sofia 100, 95123 Catania, Italy; r.chiararomano@gmail.com (R.C.R.); lucia.parafati@unict.it (L.P.); rbarbaga@unict.it (R.N.B.); giuseppe.muratore@unict.it (G.M.); 2 Radiation and Robotic S.r.l., Viale Alcide De Gasperi 103, 95024 Acireale, Italy; radiation.robotic@gmail.com

**Keywords:** ultraviolet irradiation, bakery products, *Penicillium* spp., *Saccharomycopsis fibuligera*, shelf life

## Abstract

The effect of exposure of soft wheat buns to Ultraviolet-C radiation (UV-C, 253.7 nm) was studied as an alternative to conventional treatments to control fungal spoilage and prolong shelf life. To identify the most suitable operating conditions, the study included preliminary tests on the permeability of films to UV-C irradiation, and on treatment antifungal efficacy on target microorganisms (*Penicillium digitatum* and *Saccharomycopsis fibuligera*) in Petri dishes. A 125 µm T9250B film (Cryovac^®^ Sealed Air S.r.l), commercially available for long-life bread treated with ethanol and conditioned in a modified atmosphere, was selected to pack buns before the UV-C treatment. The study was carried out along with the observation of the fungal growth of buns artificially inoculated with suspensions of *P. digitatum* and *S. fibuligera*, treated under UV-C at a distance of 25 cm between bread and the 15 W UV-C source, in comparison to untreated buns used as control. Estimation of fungal growth as well as sensory evaluation was made 2, 4, 7, 10 and 14 days after the treatment. UV-C treated buns showed a noticeable reduction of fungal spoilage and kept a tender texture for up to two weeks after packaging. UV-C treatment represents a good opportunity for the bakery industry, reducing costs and ensuring a prolonged shelf life of a commercial product, respecting the health and hedonistic expectations of the customers.

## 1. Introduction

Today’s food industry is experiencing a paradigm shift, characterized by increasing consumer demand for culinary offerings that prioritize safety and boast elevated levels of health-promoting phytochemicals. This relentless focus on health has necessitated the constant refinement of food production methods. Consequently, the focus has been on improving and replacing conventional food processing techniques such as heat treatment, sonication and radio frequency heating. Although these established methods have been proven to be effective, it is important to acknowledge their limitations, as they have the potential to adversely affect the physicochemical and nutritional properties of foods, and their implementation often involves significant energy and economic expenditures. Therefore, the need to develop innovative and sustainable food processing technologies has become increasingly urgent [1]. One such technology that has attracted considerable interest and shown remarkable promise is ultraviolet (UV) irradiation. UV light, particularly the UV-C spectrum (wavelength range: 200–280 nm), has been widely recognized for its potent antimicrobial and germicidal properties [1]. This non-thermal processing technique offers a healthy and environmentally friendly approach to ensuring food safety while preserving the integrity and nutritional value of food products. UV-C radiation is defined as ‘a non-thermal technology approved by the Food and Drug Administration (FDA) for the processing and other treatments of food that, when applied with an intensity of 1W (253.7 nm), has lethal effects for most microorganisms, including bacteria, virus, protozoa, fungi, yeasts, and algae’ [2]. UV-C irradiation has even been used for the decontamination of waste water and surfaces, and it can be used to limit the use of chemicals in the food processing chain. In the food sector, UV-C light has been used to treat fruit and vegetable juices, beverages, milk and dairy products, ready-to-eat meat products, and on the surface of high-fat foods irradiated under vacuum or in an inert atmosphere to sterilize the water used in food production and to reduce human pathogens and other microorganisms [1,2], to improve food safety and extend the shelf life of fresh produce. The germicidal effect of UV-C is to destabilize microbial DNA/RNA, resulting in altered metabolism and reproduction, and ultimately, cell death. UV-C irradiation has successfully inactivated some bacteria, yeasts and molds associated with food-borne infections and diseases and food spoilage. The use of UV-C treatment has become popular and synergistic with other technologies within hurdle technologies. Food composition and state (liquid or solid), UV-C equipment and microbial targets determine the use of UV-C treatment [1].

In accordance with Regulation (EC) No 178/2002, Article 29 (paragraph 1, letter a) [3], the European Commission requested the European Food Safety Authority (EFSA) to provide a scientific opinion on the safety assessment of UV-treated bread in the context of Regulation (EC) No 258/97. With regard to the microbiological status of the bread, which is mainly determined by the heat treatment, the EFSA experts confirmed that the application of UV treatment after baking prevents secondary microbiological contamination, for example due to the presence of mold spores in the air, which can be deposited on the surface of the product during the cooling phase. They also confirmed that irradiation does not damage the products and does not cause safety problems in terms of toxicity or allergenicity when consumed, as the treated products are equivalent to untreated products and are considered acceptable from a nutritional point of view [4].

In fact, the content of water, proteins, fats, fatty acids, carbohydrates, fibers and vitamins (mainly vitamin E and of group B) is similar between UV-treated and untreated bread, with the exception of vitamin D_2_. This is because the UV treatment applied after baking converts the ergosterol present in the bread after yeast fermentation into ergocalciferol (vitamin D_2_), mainly in the crust of the bread (in a thin layer of 2–3 mm) [5].

Under certain conditions, the UV treatment can also induce reactions between biomolecules (lipids or proteins), but it must be borne in mind that the oxidative reactions induced by the UV radiation are lower than those induced by the cooking process [6]. For this reason, scientists believe that it is comparable to conventional processes.

In recent years, this treatment has found various applications in the pharmaceutical and cosmetic fields [7,8]. It is considered a green technology that promotes environmental sustainability while preserving human health. Additionally, it can be used in the post-harvest period to preserve fruits and vegetables and replace agrochemicals and pesticides that may remain as toxic residues in food products [9]. Doses of UV-C have been tested to reduce, inactivate and retard the growth of molds and yeasts, which are food spoilage contaminants [10,11,12,13]. Some authors demonstrated [14] the potential of UV-C irradiation on the increase of some biochemical compounds of food, in support of the activation of compounds with health benefits. Furthermore, according to Chun et al. (2009), this technology could be an advantage for industrial production, reducing costs and eliminating the chemical risk, with only negligible effects on the sensory descriptors of the products [15].

It is worth noting that consumers are looking for quality and sustainable products. As color is important for consumers when choosing bakery products, researchers have tried to find suitable methods for an objective study of the shelf life. According to Grillo et al. (2014), the image analysis could be considered a valid tool to measure the shelf life trends of a bakery product in a clear, objective, reliable and non-destructive way, using modern software (KS-400 V.3.0, Carl Zeiss, Vision, Oberkochen, Germany) as a subset of sensory evaluation or other methods, following other authors who had already used it for fruits, vegetables, and meat [16].

The shelf life of bread is influenced by several factors, such as the type of flour used in its production. In fact, soft wheat bread is subject to faster staling, lower consumer acceptance and significant economic losses, and its success in the market depends on the intrinsic quality of the product and the effectiveness of the packaging and its ability to maintain the quality and limit environmental impact. Some authors have studied the possibility of reducing the thickness of materials used in thermoformed packaging and the selection of appropriate materials for gas barrier properties [17].

Given the potential limitations of conventional preservation methods, this research investigates novel technological approaches to increase the shelf life and microbial safety of bakery products. Specifically, this study aims: (i) To evaluate alternative preservation strategies that could replace or complement Modified Atmosphere Packaging (MAP) and ethanol addition, which are widely used in the bakery industry; (ii) To quantitatively assess the efficacy of short wavelength ultraviolet (UV-C) radiation in reducing microbial contamination as a preventative treatment; (iii) To maximize the shelf life of bakery products while preserving their sensory properties by developing an optimized technological protocol that strategically combines these innovative preservation techniques.

## 2. Materials and Methods

### 2.1. Determination of UV-C Irradiation Time

The UV-C treatments tested were characterized by two different times of exposure to irradiation, conventionally referred to as UV-C_T1 and UV-C_T2. UV-C_T1 corresponds to the final exposure time of 103 s, obtained from the literature on the subject (80 s) and increased by 30% to take into account the possible attenuation of the packaging thin film. UV-C_T2 corresponds to the final exposure time of 154 s, obtained by increasing the normalized UV-C_T1 exposure time of 50% [10].

### 2.2. UV-C Irradiation Test on Packaging Film

**Figure 1:**
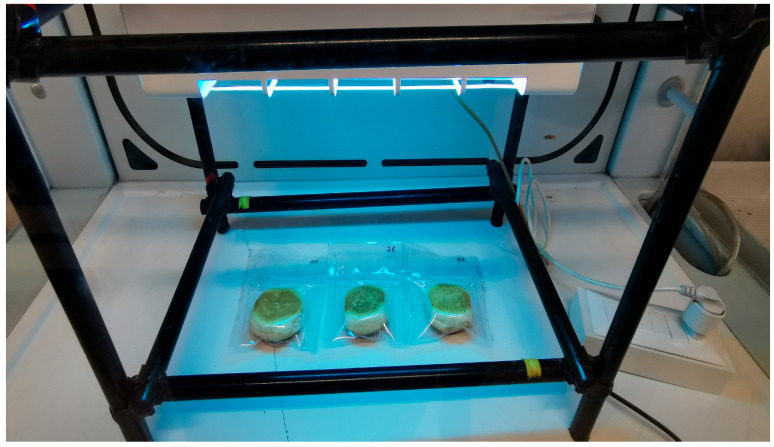
Hood and lamp used for experimental UV-C treatment.

Tests were performed to evaluate the permeability to UV-C irradiation of two films: T9250B (thickness 125 µm) and T6011B (thickness 275 µm), commercially used for long-life bread treated with ethanol and conditioned in a modified atmosphere, produced by Cryovac^®^ Sealed Air S.r.l. (I-20017 Passirana di Rho, Milano, Italy). The experiments were carried out using a UV-C lamp (Osram Puritec, HNS 36NG13, Osram—Treviso, Italy) with 15 W of irradiated power UV-C (200–280 nm) with a source-detector distance of 25 cm (referred to an experimental standard), using a precision spectroradiometer (Hopoocolor, OHSP-350 series, Hangzhou Hopoo Light & Color Technology Co., Ltd.—RPC, Hangzhou, China) positioned under the UV lamp (under the hood), essential for determining the irradiation capacity of the lamp (Figure 1).

The instrument revealed a continuous spectrum and detected a peak irradiance at 254 nm, with an initial irradiance of 1.27 mW/cm^2^ at a distance of 22 cm from the lamp and an increase in irradiance as the exposure continued. The irradiance gradually increased until it stabilized, which was achieved by switching the lamp on again. When the lamp was switched off, after one minute, the recorded value was 1.73 mW/cm^2^. Once the irradiation conditions were stabilized, the UV-C treatment could be applied to the samples. The selection of T9250B film for packaging soft wheat bread rolls was the result of preliminary irradiation tests, the results of which are shown in Table 1. The attenuation coefficient was calculated by dividing the irradiance without film by the irradiance of each film.

### 2.3. Efficacy of In Vitro UV-C Treatment on Target Microorganisms

For determining the capacity of UV-C treatment of controlling fungal spoilage of bread, preliminary Petri plate tests were carried out with *Penicillium digitatum* and *Saccharomycopsis fibuligera* strains, belonging to the Di3A collection, known for their spoilage potential of bakery products [9,10,17]. *P. digitatum* was preliminarily grown on Potato Dextrose Agar (PDA; Oxoid, CM0139B, Basingstoke, UK) at 25 °C for 5 days, while *S. fibuligera* was grown on Sabouraud Dextrose Agar (SDA; Oxoid, CM0041, Basingstoke, UK) at 25 °C for 72 h.

PDA and SDA plates were inoculated at the center with a 5 mm side plug of the target microorganisms, cut with a sterile scalpel from the edge of the actively growing fungal colony, and then subjected to UV-C treatment for 80 s without the lid in a previously sterilized closed chamber; the lid was then placed on the plates, which were incubated at 25 °C for 5 days. Plates inoculated with the two target microorganisms but not subjected to UV-C treatment were used as controls. Experiments were repeated 3 times. The reduction in radial growth of the microorganisms treated with UV-C was calculated in relation to the growth of the control (untreated) microorganisms as follows:

%I = (C − T/C) × 100,

where %I represents the inhibition of radial fungal growth, C is the radial growth measurement in the control, and T is the radial growth of the target microorganism treated with UV-C.

### 2.4. Bun Making, Artificial Contamination and UV Treatment

The soft buns were prepared in the laboratory according to a traditional recipe, without the use of preservatives, with the following ingredients for each kilogram of soft wheat flour: 385 g of water, 100 g of lard, 1.5 g of sugar, 30 g of dried yeast and 20 g of salt. All the ingredients were mixed and kneaded in a bread maker (Imetec Zero-Glu Pro, Tenacta Group SpA, Azzano S. Paolo, Bergamo, Italy), and, after shaping and leavening, baked in the maker. Baking was carried out at 150 °C for 50 min. All the soft buns had an average weight of 200 ± 10 g. *P. digitatum* and *S. fibuligera*, selected according to their frequency of occurrence on bread [18,19], were cultured on PDA for 10 days at 25 °C and on SDA for 72 h at 25 °C, respectively. Mold conidia and yeast cells were suspended in sterile physiological solution (0.9% NaCl) and counted by a Burker chamber to determine the number of cells per unit volume of the solution. Thus, the mold and yeast suspensions were adjusted to 10^6^ cells/mL with the same diluent for subsequent inoculation of the bread, which took place by pipetting 10 µL of mold or yeast suspension at four symmetrical points on the bread surface before UV-C treatment. The inoculation points were chosen to cover the whole bun, taking into account the surface area, and to ensure a homogenous distance (about 3 cm) between them and from the outer edge. After baking, the buns were removed from the bread maker and aseptically cooled in the microbiological hood at room temperature (20 ± 1 °C). Cooling took 4 h to bring the buns to room temperature, after which the buns were inoculated. Packaging was in bags (16 × 14 cm) specifically prepared for soft buns, using 125-micron T9250B internal heat-sealable film, selected on the basis of preliminary irradiation tests and taking into account the surface area of the buns.

The packaged buns were divided into 2 experimental groups (inoculated with *P. digitatum*, inoculated with *S. fibuligera*), with 3 replicates for each group, assigned with specific identification codes, resulting in a total of 18 bun samples, as shown in Table 2.

The packaged buns were treated in groups of 3, both above and below, in a hood at a distance of 25 cm from the source, with a UV-C dose of 792 mJ/cm^2^, with an exposure time of 103 s (UV-C_T1) or 154 s (UV-C_T2). After irradiation, all the previously prepared samples were placed in an incubator at 25 °C and observed at intervals of 2, 4, 7, 10 and 14 days after treatment. Fungal growth was expressed as the mean of colonies, within the respective groups inoculated with *P. digitatum* (groups 4, 5, 6) and *S. fibuligera* (groups 7, 8, 9) on the surface of the buns.

### 2.5. Sensory Evaluation

A trained panel consisting of 5 panelists performed the sensory evaluations on the bun groups described in Table 2. All evaluations took place in the Quality and Packaging Laboratory at 20 °C on the day of processing (0 d) and after 4, 7, 10 and 14 days of shelf life. Included in the study were bun samples not inoculated and not treated with UV-C (NI—NT), not inoculated and treated with UV-C_T1 (NI—UV-C_T1), and not inoculated and treated with UV-C_T2 (NI—UV-C_T2). Visual, odor, and textural attributes were identified and selected according to Dong and Karboune (2021) [20] and García-Gomez et al. (2022) [21], and the procedure followed the ISO/IEC 17025:2018 standard [22] and a descriptive method. The packaged buns were evaluated for the following descriptors: ‘crust color’ and ‘visual dryness’ with regard to the appearance; ‘overall intensity of aroma’, and ‘off-odors’ with regard to the odor. Texture was rated on ‘hardness’ and ‘softness’ measured by direct touch. The intensity scale scores ranged from 0 (no sensation) to 5 (extremely intense). The ‘hardness’ descriptor was defined as the force required to perform the first compression cycle; the ‘softness’ attribute depended on the moisture content of the crumb and its structure.

The panelists who were selected to participate in the research were informed of the general purpose of the study and signed an informed consent form, as our institution does not have an Ethics Committee for taste and food quality assessment studies.

### 2.6. Statistical Analysis

The fungal growth and sensory data for each attribute were submitted to one-way ANOVA using preservation technology as the factor. The software used to process the data was SPSS 21.0 (IBM Statistic).

## 3. Results

The efficacy of UV-C treatment for 80 s on *P. digitatum* and *S. fibuligera* inoculated, respectively, onto PDA and SDA plates by the plug method showed, after 5 days of incubation at 25 °C, a reduction in radial growth for both microorganisms. In particular, the %I was 45 for *P. digitatum* and 42 for *S. fibuligera*.

**Figure 2:**
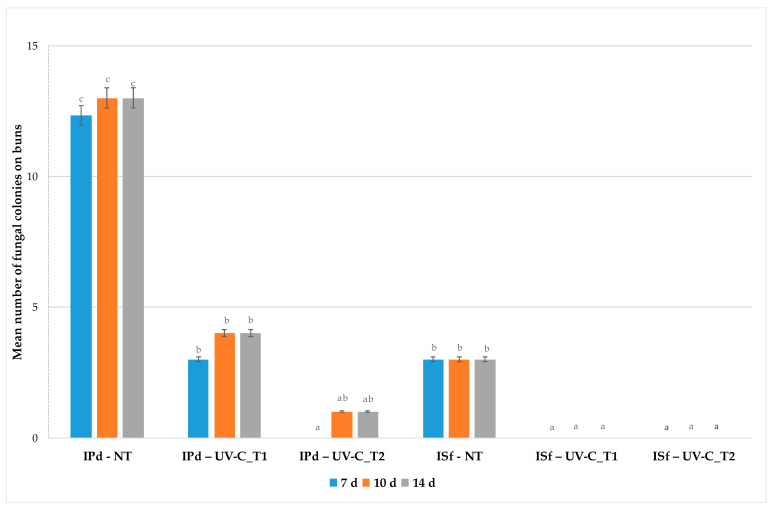
Fungal growth during shelf life in untreated and UV-C_T1/UV-C_T2-treated bun samples, artificially inoculated with *P. digitatum* and *S. fibuligera*. Data are expressed as mean ± standard deviations. Different letters indicate statistical differences between the groups for each sampling time (Tuckey’s HSD *p* < 0.90) (I = inoculated; Pd = *P. digitatum*; Sf = *S. fibuligera*; NT = Not Treated; UV-C_T1 = exposure time of 103 s; UV-C_T2 = exposure time of 154 s).

With reference to bun samples, the differences in fungal growth, expressed as the mean number of colonies on the surface of the bread, within the respective inoculated groups with *P. digitatum* and *S. fibuligera,* subjected or not to UV-C treatments, are shown in Figure 2; data are presented starting from day 7, since after 2 and 4 days of storage, no visible fungal growth was observed in all samples.

One week after the UV-C treatment, the bread samples showed great differences in fungal growth, with varying reductions in comparison with the untreated bun samples, depending on the duration of the UV-C treatment. In particular, UV-C_T1 reduced the number of *P. digitatum* colonies by 75%, while UV-C_T2 completely inhibited (100%) mold growth (100%). Comparable reduction rates of about 70% and 92% were observed for UV-C_T1 and UV-C_T2 treatments, respectively, both after 10 and 14 days of storage.

Artificial contamination with *S. fibuligera*, although present at low levels also in the UV-C untreated buns, was effectively controlled by the treatments; in fact, a 100% reduction in the number of colonies was achieved with both UV-C_T1 and UV-C_T2 treatments after 7, 10 and 14 days of storage. It is worth noting, however, that fungal growth, where present, was mainly detected on the lateral surfaces, which supports the hypothesis that the UV-C treatments were highly effective only in the areas directly exposed to radiation.

The sensory results are displayed in Table 3, starting from the 4th day after the UV-C treatments, since no statistical difference was found for any of the attributes immediately after irradiation and after 2 days of storage.

In detail, crust color was significantly increased by UV-C treatments throughout the storage period. Conversely, visual dryness was significantly reduced by UV-C treatment compared to the untreated buns. The latter finding was further confirmed by the descriptors ‘hardness’ and ‘softness’, which were significantly reduced and increased, respectively, by the UV-C treatments. With regard to the odor, the descriptor ‘overall intensity of aroma’ was significantly increased by UV-C treatments only for the last sampling time (14 days after treatment), while the descriptor ‘off-odors’ was significantly reduced by UV-C_T2 treatment in the bun samples already on the seventh day after irradiation and until the end of the storage period under consideration.

Although the sensory test used in this study does not replace traditional panel testing, this type of sensory evaluation is an effective tool for excluding any detrimental effect of UV-C treatments on the finished product.

## 4. Discussion

Foods treated with non-invasive processing methods fall under the category of ‘novel foods’ [23]. Among the non-thermal processing methods, UV irradiation, especially in the wavelength range of 200–280 nm (UV-C), is promising for extending shelf life, protecting against the growth of pathogens and spoilage microorganisms (due to its antimicrobial effects) and minimizing nutritional and sensory losses [24]. UV-C irradiation is a common alternative to pasteurization in liquid foods and beverages, given its non-toxic and non-invasive action [25]. It is also used on vegetable-based nonsolid food products [14]. However, its effectiveness is limited by its lack of penetrating capacity. Additionally, the antimicrobial effect of UV-C irradiation depends on the UV-C dose, the characteristics of the microbial target and the characteristics of the treated food [14]. UV-C treatment of foods must be properly designed to ensure safety, and to extend shelf life [26], while considering the attenuation of the radiation by food packaging materials. UV-C cannot penetrate most synthetic polymers or conventional glass [27,28], and, in this study, it was essential to test the attenuation of the radiation by the films studied beforehand and to select the one with the higher irradiance before proceeding with the treatment of the packaged buns.

The 125 µm film was more transmissive to UV-C light than the 275 µm film, possibly due to the thickness of the film, but it is unclear how the material will perform under long-term exposure to UV-C irradiation, as some authors have reported changes and degradation of the polymers under UV-C exposure [28].

The growth of both yeast and mold in UV-C treated Petri plates indicates that the microorganisms were not completely eliminated, but reduced. However, the colony diameter of both target microorganisms in the treated plates was reduced compared to the untreated ones, as a result of the reduction of initially viable cells caused by DNA damage, protein polymerization, enzyme inactivation and increase in cell membrane permeability or by prolongation of the growth lag phase. Such findings are in accordance with previous studies that demonstrated how UV-C treatment is able to reduce the viability of fungal spores, but with efficacy depending on inoculation methods, fungal genus and irradiation exposure methods [10,29].

*P. digitatum* was more sensitive to UV-C treatment than *S. fibuligera*, confirming that the microbial resistance to irradiation, as the ability to repair ultraviolet cell damage, is dependent on fungal species and on strain, although to the best of our knowledge, there are no studies evaluating the effects of UV-C treatment to reduce the growth of *S. fibuligera*. Higher doses or longer exposure times may be required to obtain a product free from environmental fungal contamination [28]. The antifungal efficacy of the UV-C treatment was evident both in Petri plates, where a reduction in fungal colony diameter was observed, and on the yeast- and mold-inoculated soft buns, where a restrained growth of the artificially inoculated microorganisms was observed from the third observation (7 d) onwards.

In view of the results, the efficacy of the treatment studied can be considered ‘temporary’ at the dose applied, but certainly effective in delaying fungal spoilage.

Shelf life and growth of *P. digitatum* may depend on the fluence of UV-C irradiation. As studied by Debonne et al. (2023), a higher fluence of UV-C irradiation increased the shelf life of different types of bread, and potential contamination of bread after UV-C treatment may affect with the safety of the product [28]. On the one hand, a higher fluence or intensity of irradiation may be more effective in killing microorganisms, but may produce off-flavors and negative aromas, although little research has been conducted on this subject [29,30], and, if excessive, irradiation may be harmful to human health and damaging to surfaces [14].

In this study, all samples showed the presence of microbial contamination one week after UV-C irradiation, almost exclusively on the lateral surfaces, also called ‘shaded areas’, which were not directly exposed to UV-C irradiation. The presence of *S. fibuligera* and *P. digitatum* colonies on the side of the buns could be due to the static system used, and although the buns were turned to achieve a wider decontamination, the treatment was not fully effective. Multi-sided exposure systems could provide food with a deep decontamination on the top and bottom [28], and longer exposure times should be used to achieve a complete elimination of microorganisms.

It is also important to highlight that all samples treated with UV-C remained soft up to 2 weeks after treatment and had a higher aroma intensity and a lower score for the off-odor descriptor. The effects of irradiation on the rheological properties of the buns are unclear, considering that the treated buns retained their texture throughout the expected shelf life, and require further study.

## 5. Conclusions

On the basis of this preliminary study, which focused on the application of an innovative methodology based on UV radiation to extend the shelf life of bakery products, it can be demonstrated that new approaches can replace traditional technologies, such as the use of ethanol and modified atmospheres, commonly used in the bakery sector to preserve the sensory characteristics of products. UV radiations, particularly in the UV-C wavelength range, have been used to evaluate the effectiveness of this treatment in reducing the growth of microorganisms that are common spoilage agents in baked products: *P. digitatum* and *S. fibuligera*. The use of UV-C treatment to prolong shelf life and reduce the growth of these fungal species, which contaminate most bakery products, should be well designed and provide a system consisting of multiple lamps or a rotating system that allows the radiation to reach every point of the food, especially when it is a solid food with a complex and uniform structure. Radiation attenuation and the effects of UV-C exposure on both the food and the packaging material are important considerations in system design to ensure system efficiency.

## Figures and Tables

**Table 1:** Values of UV-C irradiance (mW/cm^2^) and attenuation coefficient of films.

	Without Film	T9250B Film (Thickness 125 µm)	T6011B Film (Thickness 275 µm)
Irradiance (mW/cm^2^)	1.73	1.27	1.10
Attenuation coefficient	0.00	1.30	1.51

**Table 2:** Distribution of bun samples by treatment and inoculum type (I = inoculated; Pd = *P. digitatum*; I = *S. fibuligera*; NT = Not Treated; UV-C_T1 = exposure time of 103 s; UV-C_T2 = exposure time of 154 s).

Inoculated with *P. digitatum*		Inoculated with *S. fibuligera*	
IPd—NT	4A, 4B, 4C	ISf—NT	7A, 7B, 7C
IPd—UV-C_T1	5A, 5B, 5C	ISf—UV-C_T1	8A, 8B, 8C
IPd—UV-C_T2	6A, 6B, 6C	ISf—UV-C_T2	9A, 9B, 9C

**Table 3:** Sensory data of UV-C_T1- and UV-C_T2-treated bun samples after 4, 7, 10 and 14 days from the UV-C treatment (I = inoculated; Pd = *P. digitatum*; Sf = *S. fibuligera*; NI = Not Inoculated; NT = Not Treated; UV-C_T1 = exposure time of 103 s; UV-C_T2 = exposure time of 154 s).

Samples	Storage Time (d)	Crust Color	Visual Dryness	Overall Intensity of Aroma	Off-Odors	Hardness	Softness
NI-NT	4	4.00 ± 0.00 a	0.80 ± 0.45 b	4.20 ± 0.45	0.00 ± 0.00 a	1.00 ± 0.00 b	3.40 ± 0.89 a
NI—UV-C_T1		5.00 ± 0.00 b	0.00 ± 0.00 a	4.80 ± 0.45	0.00 ± 0.00 a	0.00 ± 0.00 a	5.00 ± 0.00 b
NI—UV-C_T2		5.00 ± 0.00 b	0.00 ± 0.00 a	5.00 ± 0.00	0.00 ± 0.00 a	0.00 ± 0.00 a	5.00 ± 0.00 b
IPd-NT		4.60 ± 0.55 ab	0.40 ± 0.55 ab	4.40 ± 0.55	0.20 ± 0.35 a	1.60 ± 0.55 b	4.20 ± 0.84 ab
IPd—UV-C_T1		4.80 ± 0.45 ab	0.00 ± 0.00 a	4.80 ± 0.45	0.20 ± 0.45 a	0.00 ± 0.00 a	5.00 ± 0.00 b
IPd—UV-C_T2		4.80 ± 0.45 ab	0.00 ± 0.00 a	4.80 ± 0.45	0.20 ± 0.45 a	0.00 ± 0.00 a	5.00 ± 0.00 b
ISfd-NT		4.40 ± 0.55 ab	0.60 ± 0.55 ab	4.20 ± 1.09	0.20 ± 0.45 a	1.20 ± 0.84 b	4.20 ± 0.45 ab
ISf— UV-C_T1		4.80 ± 0.45 ab	0.20 ± 0.45 ab	4.60 ± 0.55	0.20 ± 0.45 a	0.00 ± 0.00 a	500 ± 0.00 b
ISf— UV-C_T2		4.80 ± 0.45 ab	0.00 ± 0.00 a	4.80 ± 0.45	0.20 ± 0.45 a	0.00 ± 0.00 a	5.00 ± 0.00 b
	**Significance**	*	***	n.s.	n.s.	***	***
NI-NT	7	3.40 ± 0.55 a	1.20 ± 0.45 bc	4.20 ± 0.45	1.40 ± 0.55 b	2.60 ± 0.55 d	1.60 ± 0.55 a
NI—UV-C_T1		4.20 ± 0.55 ab	1.00 ± 0.00 abc	4.60 ± 0.55	0.60 ± 0.55 ab	0.80 ± 0.45 ab	4.40 ± 0.55 c
NI—UV-C_T2		4.60 ± 0.45 b	0.80 ± 0.45 abc	4.80 ± 0.45	0.20 ± 0.45 a	0.40 ± 0.55 a	4.60 ± 0.55 c
IPd-NT		4.20 ± 0.45 ab	1.40 ± 0.55 c	4.00 ± 0.71	1.20 ± 0.45 ab	2.00 ± 0.71 bcd	2.80 ± 0.45 b
IPd—UV-C_T1		4.60 ± 0.55 b	0.20 ± 0.45 a	4.40 ± 0.55	0.60 ± 0.55 ab	0.60 ± 0.55 a	4.40 ± 0.55 c
IPd—UV-C_T2		4.80 ± 0.45 b	0.20 ± 0.45 a	4.60 ± 0.55	0.20 ± 0.45 a	0.20 ± 0.45 a	4.60 ± 0.55 c
ISfd-NT		4.20 ± 0.45 ab	0.80 ± 0.45 abc	4.00 ± 0.00	0.80 ± 0.45 ab	2.20 ± 0.84 cd	3.80 ± 0.45 bc
ISf— UV-C_T1		4.60 ± 0.55 b	0.40 ± 0.55 ab	4.40 ± 0.55	0.40 ± 0.55 ab	1.00 ± 0.71 abc	4.00 ± 0.00 c
ISf— UV-C_T2		4.80 ± 0.45 b	0.20 ± 0.45 a	4.80 ± 0.45	0.20 ± 0.45 a	0.80 ± 0.45 ab	4.60 ± 0.55 c
	**Significance**	**	***	n.s.	**	***	***
NI-NT	10	3.20 ± 0.45 a	2.00 ± 0.00 c	3.80 ± 0.45 a	1.60 ± 0.55 b	2.60 ± 0.55 b	1.40 ± 0.55 a
NI—UV-C_T1		4.00 ± 0.71 ab	1.20 ± 0.45 abc	4.20 ± 0.45 a	1.00 ± 0.00 ab	1.40 ± 0.55 a	4.00 ± 0.00 d
NI—UV-C_T2		4.40 ± 0.55 b	1.00 ± 0.00 ab	4.40 ± 0.55 a	0.60 ± 0.55 ab	1.00 ± 0.00 a	4.20 ± 0.45 d
IPd-NT		4.00 ± 0.00 a	1.60 ± 0.55 bc	3.60 ± 0.55 a	1.40 ± 0.55 ab	3.20 ± 0.45 b	2.40 ± 0.55 ab
IPd—UV-C_T1		4.40 ± 0.55 ab	0.60 ± 0.55 a	4.40 ± 0.55 a	0.80 ± 0.45 ab	1.40 ± 0.55 a	3.40 ± 0.89 bcd
IPd—UV-C_T2		4.60 ± 0.55 b	0.40 ± 0.55 a	4.60 ± 0.55 a	0.60 ± 0.55 ab	1.20 ± 0.45 a	3.80 ± 0.45 cd
ISfd-NT		4.00 ± 0.00 a	1.20 ± 0.45 abc	3.80 ± 0.45 a	1.60 ± 0.55 b	3.20 ± 0.84 b	2.60 ± 0.89 abc
ISf— UV-C_T1		4.40 ± 0.55 ab	0.80 ± 0.45 ab	4.40 ± 0.55 a	0.80 ± 0.45 ab	1.00 ± 0.00 a	3.80 ± 0.45 cd
ISf— UV-C_T2		4.60 ± 0.55 b	0.40 ± 0.55 a	4.60 ± 0.55 a	0.40 ± 0.55 a	0.60 ± 0.55 a	4.40 ± 0.55 d
	**Significance**	**	***	n.s.	***	***	***
NI-NT	14	2.20 ± 0.45 a	2.80 ± 0.45 d	3.00 ± 0.00 a	1.80 ± 0.45 bc	4.20 ± 0.84 cd	0.80 ± 0.84 a
NI—UV-C_T1		3.80 ± 0.45 bc	1.40 ± 0.89 abc	4.00 ± 0.00 b	0.80 ± 0.45 ab	1.20 ± 0.45 ab	3.80 ± 0.84 b
NI—UV-C_T2		3.60 ± 0.55 bc	1.20 ± 0.45 ab	4.20 ± 0.45 b	0.40 ± 0.55 a	1.00 ± 0.71 a	4.00 ± 0.00 b
IPd-NT		3.40 ± 0.55 b	2.20 ± 0.45 cd	3.20 ± 0.45 a	2.00 ± 0.00 c	3.80 ± 0.45 cd	1.60 ± 0.55 a
IPd—UV-C_T1		4.00 ± 0.00 bc	1.00 ± 0.00 a	4.20 ± 0.45 b	1.20 ± 0.84 abc	2.40 ± 0.55 bc	3.60 ± 0.55 b
IPd—UV-C_T2		4.40 ± 0.55 c	0.80 ± 0.45 a	4.20 ± 0.45 b	0.80 ± 0.45 ab	2.40 ± 0.55 bc	3.80 ± 0.45 b
ISfd-NT		3.80 ± 0.45 bc	2.00 ± 0.00 bcd	2.80 ± 0.45 a	2.20 ± 0.45 c	4.20 ± 0.45 cd	1.20 ± 0.45 a
ISf— UV-C_T1		4.20 ± 0,45 bc	1.00 ± 0.00 a	4.00 ± 0.00 b	1.20 ± 0.45 abc	2.00 ± 0.71 ab	3.60 ± 0.55 b
ISf— UV-C_T2		4.40 ± 0.55 c	0.80 ± 0.45 a	4.20 ± 0.45 b	0.80 ± 0.84 ab	3.60 ± 0.55 cd	4.20 ± 0.45 b
	**Significance**	***	***	***	***	***	***

Data are expressed as mean ± standard deviations. Different letters indicate statistical differences between the same column and for each sampling time. *** Significance for *p* ≤ 0.001; ** Significance for *p* ≤ 0.01; * Significance for *p* ≤ 0.05; n.s. not significant.

## Data Availability

The original contributions presented in the study are included in the article, further inquiries can be directed to the corresponding authors.
